# Periconceptional maternal dairy-rich dietary pattern is associated with prenatal cerebellar growth

**DOI:** 10.1371/journal.pone.0197901

**Published:** 2018-05-23

**Authors:** Francesca Parisi, Melek Rousian, Irene V. Koning, Sten P. Willemsen, Jeanne H. M. de Vries, Eric A. P. Steegers, Régine P. M. Steegers-Theunissen

**Affiliations:** 1 Department of Obstetrics and Gynecology, Erasmus MC, University Medical Center, Rotterdam, The Netherlands; 2 Department of Biostatistics, Erasmus MC, University Medical Center, Rotterdam, The Netherlands; 3 Division of Human Nutrition, Wageningen University, Wageningen, The Netherlands; 4 Department of Pediatrics, Division of Neonatology, Erasmus MC, University Medical Center, Rotterdam, The Netherlands; The University of Manchester, UNITED KINGDOM

## Abstract

**Background:**

Maternal nutrition during pregnancy has been related to intrauterine brain development and neurodevelopmental disabilities in adult life. We aim to investigate associations between periconceptional maternal dietary patterns and prenatal cerebellar growth from the first trimester onwards.

**Materials and methods:**

126 women with singleton non-malformed pregnancies were enrolled before 8 weeks of gestation in the Rotterdam periconceptional cohort between 2013 and 2015. Periconceptional maternal dietary patterns were extracted from food frequency questionnaires and associated with blood biomarkers and micronutrient intakes. Serial two-dimensional and three-dimensional ultrasound scans were performed at 9, 11, 22, 26 and 32 weeks of gestation for transcerebellar diameter (TCD) measurement. Linear mixed models were estimated to investigate associations between periconceptional maternal dietary patterns and longitudinal TCD measurements as a function of gestational age.

**Results:**

We performed a median of 4 scans per pregnancy, resulting in 570 total datasets. The success rate of TCD measurements was 87% (range 65–100%), depending on gestational age. The Mediterranean, Western, egg-rich and dairy-rich dietary patterns were extracted, explaining 37.2% of the overall variance of food intake in this population. The dairy-rich dietary pattern was positively associated with cerebellar growth trajectories (β = 0.02 (95% CI: 0.01; 0.03) √mm, p = 0.01). Maternal strong adherence to this dietary pattern increased TCD measurements by 0.8 standard deviation scores (SDs) compared to weak adherence, reflected in increased TCD estimates of 0.44 mm at 9 weeks (+6.8%), 0.88 mm at 22 weeks (+3.6%), and 1.17 mm at 32 weeks (+2.8%). No significant associations were detected for the Mediterranean, Western and egg-rich dietary patterns.

**Conclusions:**

This study shows a positive association between periconceptional maternal adherence to a dairy-rich dietary pattern and human prenatal TCD measurements as a proxy of cerebellar growth. Next step is the investigation of the impact on neurodevelopmental outcomes in the offspring.

## Introduction

The traditional paradigm of the cerebellum exclusively as a part of the motor system has been progressively abandoned in the last decades. It is now clear that the cerebellum plays a crucial role in human acquisition of cognitive and emotional attributes and abilities [1, 2]. Starting in the first weeks of pregnancy with the development of the Purkinje cells, cerebellar development continues during the third trimester of pregnancy and even postnatally when the cerebellum outgrows its dimension more than three times [2, 3]. This prolonged process explains the wide window of cerebellum sensitivity to environmental insults. Several prenatal exposures have been related to impaired fetal cerebellar growth, including maternal alcohol consumption, smoking and toxin exposures [4, 5]. Moreover, animal models showed significant associations between nutritional exposures during pregnancy and impaired intrauterine cerebellar development, reporting altered growth of Purkinje cells in case of iodine deficiency, modified methylation profiles after folic acid supplementation and increased cerebellar lipoperoxidation among low protein-fed offspring [6, 7]. No human data are available on the associations between maternal dietary patterns and prenatal cerebellar development, despite the well-known associations with neurodevelopmental outcomes [8–10].

The introduction of high frequency transvaginal and transabdominal probes and three-dimensional ultrasound (3D US) has dramatically improved the chance of evaluating human embryonic and fetal structures, and reference curves of cerebellar growth are now available as early as the embryonic period [11].

In the current study, we aim to investigate associations between periconceptional maternal dietary patterns and prenatal cerebellar growth trajectories measured by longitudinally collected transcerebellar (TCD) diameters from the first trimester onwards.

## Materials and methods

This study was conducted at the Department of Obstetrics and Gynecology, Erasmus MC, University Medical Center in Rotterdam, the Netherlands, in the setting of the ongoing Rotterdam Periconception cohort (Predict Study) [12]. The protocol was approved by the Medical Ethical and Institutional Review Board at the Erasmus MC, University Medical Center in Rotterdam, the Netherlands, and all participants signed a written informed consent on behalf of themselves and the unborn child (METC Erasmus MC 2004–277).

Eligible for the current study were all women aged at least 18 years old, with an intrauterine singleton viable pregnancy before 8^+0^ weeks of gestation between November 2013 and March 2015. We selected pregnancies conceived spontaneously, through intrauterine insemination (IUI) or assisted reproductive technologies using homologous oocyte(s). Ongoing pregnancies complicated with congenital anomalies were excluded from the analysis. Missing or unreliable periconceptional maternal nutritional evaluations were additional exclusion criteria. The pregnancy dating procedure was based on crown-rump length (CRL) measurements before 13 weeks of gestation for spontaneous and IUI pregnancies, on the date of oocyte retrieval plus 14 days for *in vitro* fertilization (IVF) and intracytoplasmic sperm injection (ICSI) derived pregnancies, or from the day of embryo transfer plus 17–18 days for cryopreserved transfers [13].

At enrolment, baseline anthropometric measurements and general information on age, geographical origin, educational level, obstetric and medical anamnesis and periconceptional exposures (smoking, alcohol use, folic acid or multivitamin supplement use) were collected. Data on birth outcomes were collected from medical records (neonatal gender, birth weight, gestational age at birth).

The semi-quantitative food frequency questionnaire (FFQ) developed by the division of Human Nutrition, Wageningen University, the Netherlands, and validated for women of reproductive age, was used at recruitment to assess maternal nutritional intake over the previous month [14, 15]. Participants were asked to report the frequency of consumption, portion size and preparation method of 196 food items structured according to meal patterns. Average daily nutritional values were calculated using the Dutch food composition table [16] and reports with implausible energy intakes were considered unreliable and therefore excluded from the analysis. Based on similar origin and nutrient contents, the 196 FFQ food items were condensed into 23 food groups and dietary patterns were derived using principal component analysis (PCA) as extensively described by Hoffmann [17, 18]. PCA automatically assigns all participants a component score for each dietary pattern representing their adherence to that specific dietary pattern, meaning that every woman possibly shows high adherence (component scores> 0) for two or more dietary patterns at the same time. One fasting venous blood sample for serum and red blood cell (RBC) folate, serum vitamin B12 and plasma total homocysteine (tHcy) determination was collected at enrolment and the laboratory procedures have been extensively described elsewhere [19].

All women underwent longitudinal ultrasound examinations performed by one trained sonographer for TCD measurements (IVK). First trimester 3D US examinations were performed using a high-resolution transvaginal probe (6–12 MHz) of the Voluson E8 system (GE Medical Systems, Zipf, Australia). All 3D volumes were stored as Cartesian and 4DView volumes and TCD measurements were performed offline using 4D View Version 5.0 (GE Medical Systems). Due to the low success rate of cerebellar measurements performed before 8 weeks of gestation, only 3D US volumes at 9 weeks of gestation or later were included in the analysis [11]. The technique, reliability and protocol used for cerebellar measurements at 9 and 11 weeks of gestation have been previously validated and extensively described by our group [11, 20]. Briefly, first trimester cerebellar measurements were performed manually using a 4D view software in a coronal section of the head through the rhombencephalon, showing the cerebellum, fourth ventricle, and plexus choroideus of the fourth ventricle. An excellent intra- and inter-observer reliability was reported, with intraclass correlation coefficients above 0.98. First trimester cerebellar growth curves and associations with gestational age and CRL were determined in the same population [11]. Second and third trimester ultrasound scans were performed at 22, 26 and 32 weeks of gestation using 1–7 MHz transabdominal transducer or a 6–12 MHz transvaginal transducer of the Voluson E8 system. Standardized 2D biometric measurements of the TCD were obtained online by slightly rotating the transducer from the transverse cross-section of the thalamic plane to the posterior fossa, enabling the ‘outer-to-outer’ measurement of the cerebellum, as recommended by the ISUOG guidelines. All embryonic and fetal TCD measurements were performed three times by the same researcher (IVK) and the mean values were used for the analysis.

Finally, intrauterine growth trajectories according to estimated fetal weight, birth weight and corresponding percentiles were considered in order to detect abnormal fetal growth trajectories.

### Statistical analyses

All analyses were performed using IBM SPSS version 21.0 (Armonk, NY: IBM Corp) and R version 3.2.1 (The R Foundation for Statistical Computing). P-values ≤0.05 were considered significant. Maternal characteristics and dietary pattern adherence (component scores) were compared between excluded and included pregnancies using Chi-square or exact tests for ordinal variables and Mann-Whitney U test for continuous variables. The basal metabolic rate (BMR) was calculated using the new Oxford equations stratified by age and gender, as described and validated elsewhere [21]. Finally, the food intake level (FIL) was obtained as the ratio of energy intake divided by BMR and compared with the physical activity level (PAL) of a sedentary lifestyle (cut off 1.35) to estimate underreporting. We compared serum and RBC folate, vitamin B12, and tHcy concentrations between women with strong (component scores >0) and weak (component scores <0) adherence to each dietary pattern using the Mann-Whitney U test. Univariable linear regression was performed to investigate correlations between nutrient intake and the extracted dietary patterns.

To investigate associations between periconceptional maternal dietary patterns and repeated cerebellar measurements, we estimated linear mixed models using TCD measurements as response variable and gestational age as predictor. This analysis allows the longitudinal modeling of repeated TCD measurements, taking into account the correlation between serial measurements within the same pregnancy. Square root transformation of TCD data resulted in approximate normal distribution of observations with a constant variance, as required by linear mixed models, and linearity with gestational age. A random intercept and slope were used to model the within subject correlation. An interaction with gestational age did not improve the model fit, suggesting a constant and continuous effect of dietary patterns on longitudinal TCD measurements. First, we estimated a mixed model adjusted for energy intake (model 1). Model 2 depicts the estimates including additional adjustment for potential confounding factors (parity, alcohol use, smoking, folic acid/multivitamin supplement use, age, BMI, conception mode and geographical origin). TCD variance expressed as standard deviation score (SDs) over gestation was calculated from model 1 as follows: SDs = TCD(x-mean)/SD to compare the effect of maternal strong versus weak adherence to the dietary patterns significantly associated with cerebellar growth. In order to evaluate the effect of maternal adherence to the dietary patterns in different trimesters of pregnancy and provide a clear interpretation from a clinical perspective, longitudinal TCD measurements were further transformed back to the original scale and plotted against gestational age by comparing maternal strong *versus* weak adherence to the dietary patterns significantly associated with cerebellar measurements. Finally, due to the association between preterm birth, intrauterine fetal growth and brain development, we performed a subgroup analysis excluding all pregnancies complicated by preterm birth, intrauterine growth restriction and low birth weight.

## Results

A total of 126 viable pregnancies out of 227 were included in the analysis, after exclusion of pregnancies achieved after egg cell donation (n = 3), missing or unreliable periconceptional FFQs (n = 94), fetal or neonatal congenital anomalies (n = 3) and missing ultrasound data (n = 1). Table 1 shows maternal baseline characteristics and the sensitivity analysis comparing included and excluded pregnancies. Included and excluded pregnancies were comparable for all characteristics, except for folic acid supplement use.

**Table 1:** Maternal baseline characteristics of the study population and excluded pregnancies.

Maternal characteristics	Study population (n = 126)	M	Excluded pregnancies (n = 100)	M	p-value
Maternal age, y median (range)	32 (22–45)	4	31 (21–48)	4	0.45
Nulliparous, n (%)	31 (24.8)	1	34 (35.8)	5	0.08
Geographical origin		2		4	0.55
Western, n (%)	98 (79.0)		79 (82.3)		
Non Western, n (%)	26 (21.0)		17 (17.7)		
Educational level		2		0	0.89
High, n (%)	60 (48.4)		46 (49.5)		
Intermediate, n (%)	50 (40.3)		35 (37.6)		
Low, n (%)	14 (11.3)		12 (12.9)		
BMI, kg/m^2^ median (range)	24.1 (16.3–39.7)	0	23.1 (15.2–43.4)	5	0.83
Alcohol use, n (%)	36 (29.3)	3	29 (30.9)	6	0.80
Periconception smoking, n (%)	18 (14.5)	2	17 (17.2)	1	0.59
Periconception folic acid/multivitamin use, n (%)	115 (92.7)	2	79 (84.0)	6	**0.04**
Mode of conception IVF/ICSI, n (%)	32 (25.4)	0	25 (25.0)	0	0.95

The comparison among groups was performed using Chi-square or exact tests for ordinal variables and Mann-Whitney *U* test for continuous variables.

M = missing values, BMI: body mass index, IVF: in vitro fertilization, ICSI: intracytoplasmic sperm injection.

**Fig 1:**
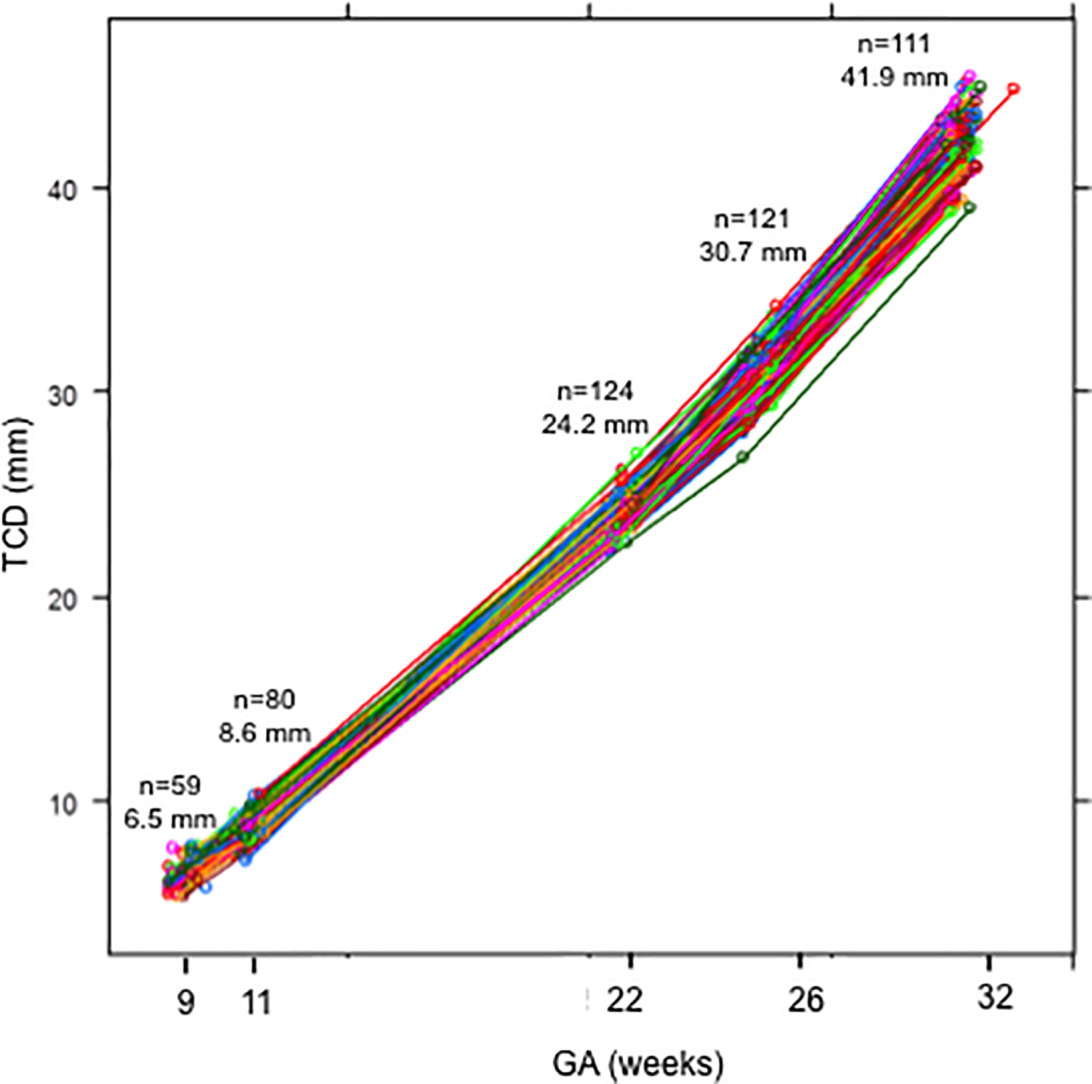
Longitudinal transcerebellar diameter (TCD, mm) measurements in the study population. Mean values at 9, 11, 22, 26 and 32 weeks of gestation are reported. Dots and lines represent TCD measurements and trajectories of a single included pregnancy. n: number; GA: gestational age.

Median gestational age at birth was 272 days (range 182–296), birth weight was 3293 g (range 665–4380) and 50.4% of newborns were males. Preterm births (<37 weeks of gestation) counted a total of 10 pregnancies, including 6 intrauterine growth restricted fetuses, whereas one pregnancy ended with a low birth weight baby (LBW, birth weight <2500 g), thus leading to a total of 115 term pregnancies with normal intrauterine growth trajectory and birth weight. A median of four scans per pregnancy (range 1–5) was performed, resulting in a total of 570 datasets. The success rate of TCD measurements was 86.8%, depending on gestational age (71.1% at 9 weeks, 65% at 11 weeks, 100% at 22 weeks, 98% at 26 weeks and 95% at 32 weeks of gestation). Fig 1 shows cerebellar growth trajectories depicted by longitudinal TCD measurements in the study population.

Based on the scree plot and interpretability of the extracted patterns, four major components (dietary patterns) with eigenvalues above 1.5 were identified, explaining 37.2% of the overall variance in dietary intake (Table 2). We labeled the first component ‘Mediterranean’ because of high loadings on vegetables, olive oil, fish, fruit, nuts and legumes, and relatively low loading on meat. The second component included high intakes of meat, soups, snacks, mayonnaise, potatoes and sauces, and low intake of fruit, resembling the ‘Western’ dietary pattern. The third component was highly associated with eggs, nuts, fish, sauces and non-alcoholic beverage consumption and was labeled ‘egg-rich’. The fourth component yielded a dietary pattern high in the consumption of dairy products, fruit, cereals, non-alcoholic beverage and whole grain and was labeled ‘dairy-rich’.

**Table 2:** Relation between food groups and extracted dietary patterns expressed by factor loadings.

Food group	Mediterranean	Western	Egg-rich	Dairy-rich
**Variance explained (%)**	11.9	10.2	8.2	6.9
**Butter**	,066	,178	,048	,112
**Cereals**	-,051	,041	,036	,480
**Dairy**	-,094	,036	-,049	,832
**Eggs**	,094	,067	,782	-,095
**Fat Liquid**	-,448	,188	,209	-,015
**Other fats**	-,051	,056	-,052	,078
**Fish**	,279	-,276	,278	-,042
**Fruits**	,241	-,030	,129	,603
**Legumes**	,732	,116	,137	-,019
**Margarine**	-,199	-,013	-,138	,222
**Mayonnaise**	,147	,702	-,004	-,127
**Meat**	-,024	,750	-,130	,142
**Non-alcoholic**	-,238	-,133	,603	,272
**Nuts**	,378	-,044	,584	,109
**Potatoes**	-,038	,267	,140	-,027
**Refined Grains**	,022	-,058	,019	,034
**Sauces**	,104	,330	,379	-,006
**Snacks**	,066	,438	,158	-,052
**Soup**	-,004	,669	,119	-,027
**Sugars**	-,002	,211	-,053	-,018
**Vegetables**	,760	,088	,095	,188
**Olive oil**	,665	-,006	-,061	-,104
**Whole grains**	,020	-,254	-,096	,395

The factor loadings describe how strong the association between the food groups and each of the extracted dietary patterns is. The factor loadings with the highest absolute value were used for labeling.

Women with strong adherence to the Mediterranean dietary pattern (component scores> 0) showed significantly higher serum and RBC folate concentrations compared to women with weak adherence (component scores< 0) to the same dietary pattern (serum folate: 43.1 (range 16.4–223.0) and 34.6 (range 12.6–451.3) nmol/l, p<0.001; RBC folate: 1429.0 (range 634.0–2256.0) and 1244 (range 466–2919) nmol/l, p = 0.02 respectively). Strong adherence to the egg-rich dietary pattern was associated with significantly higher serum and RBC folate concentrations compared to weak adherence (serum folate: 41.4 nmol/l (range 14.8–223.0) and 34.8 nmol/l (range 12.6–451.3), p = 0.02; RCB folate: 1468 nmol/l (range 866–2256) and 1258 nmol/l (range 466–2919), p = 0.01 respectively). Strong adherence to the dairy-rich dietary pattern was associated with significantly lower tHcy concentrations compared to weak adherence (5.9 μmol/l (range 3.4–11.3) and 6.5 μmol/l (range 3.7–31.4), p = 0.02). Adherence to the Western dietary pattern was not associated with the investigated biomarker concentrations.

S1 Table shows maternal baseline characteristics according to high adherence to the extracted dietary patterns. Table 3 shows the correlation coefficients for nutrient intake and the extracted dietary patterns. Maternal intake of all investigated micronutrients was significantly and positively correlated to the dairy-rich dietary pattern, except for the omega-3 fatty acid intake. The mean FIL of the study population was 1.38.

**Table 3:** Correlations between nutrient intake and the four extracted dietary patterns expressed by correlation coefficients.

Nutrient intake	Mediterranean	Western	Egg-rich	Dairy-rich
ALA	0.30 **	0.41**	0.32 **	0.20 *
EPA	0.30 **	-0.10	0.41 **	0.02
DHA	0.35 **	-0.13	0.32 **	0.02
Proteins	0.22 *	0.58**	0.15	0.48 **
Fats	0.24 **	0.62 **	0.18 *	0.20 *
Fiber	0.50 **	0.09	0.17	0.53 **
Vitamin B6	0.14	0.35 **	0.21 *	0.55 **
Vitamin B12	0.19 *	0.56 **	0.10	0.27 **
Folate	0.38 **	0.17	0.13	0.46 **
Zink	0.23 **	0.57 **	0.16	0.52 **
Vitamin A	-0.04	0.69 **	-0.05	0.14 *
Vitamin B1	0.16	0.61 **	0.15	0.46 **
Vitamin B2	0.16	0.39 **	0.11	0.72 **
Vitamin C	0.31 **	0.18 *	0.14	0.38 **
Vitamin E	0.28 **	0.40 **	0.24 **	0.18 *

Univariable linear regression was performed to evaluate correlations between nutrient intake and dietary patterns

* p<0.05

** p<0.01.

ALA: alpha-linolenic acid; EPA: eicosapentaenoic acid; DHA: docosahexaenoic acid.

Table 4 shows the effect estimates from linear mixed models in the study population. No significant associations were detected between the Mediterranean, Western, egg-rich dietary patterns and cerebellar growth trajectories, for both model 1 and 2. Conversely, maternal adherence to the dairy-rich dietary pattern was associated with increased TCD measurements for both model 1 (β = 0.02 √mm (95% CI: 0.01; 0.03), p<0.01) and 2 (β = 0.02 √mm (95% CI: 0.01; 0.03), p<0.01), leading to a difference on TCD variance of about 0.8 SDs over gestation in case of strong *versus* weak adherence to the dietary pattern. Additional adjustment for fetal gender did not modify the detected associations.

**Table 4:** Effect estimates from linear mixed models for the associations between periconceptional maternal dietary patterns and embryonic and fetal cerebellar growth trajectories, as a function of gestational age.

	EFFECT ESTIMATES TCD β (95%CI), √mm			
Dietary pattern	Model 1	p-value	Model 2	p-value
**Mediterranean**	0.00 (-0.01; 0.01)	0.78	0.00 (-0.01; 0.02)	0.94
**Western**	-0.01 (-0.02; 0.00)	0.17	-0.01 (-0.02; 0.01)	0.44
**Egg-rich**	-0.00 (-0.01; 0.01)	0.86	0.00 (-0.01; 0.01)	0.99
**Dairy-rich**	0.02 (0.01; 0.03)	**<0.01**	0.02 (0.01; 0.03)	**<0.01**

Effect estimates represent the amount of change in square root transcerebellar diameter (TCD, **√**mm) per unit of increase in component score. Model 1 is adjusted for energy intake. Model 2 is adjusted for additional potential confounding factors (energy intake, conception mode, alcohol, smoke, parity, age, BMI, folic acid supplement/multivitamin use, geographical origin).

TCD: transcerebellar diameter; CI: confidence interval.

**Fig 2:**
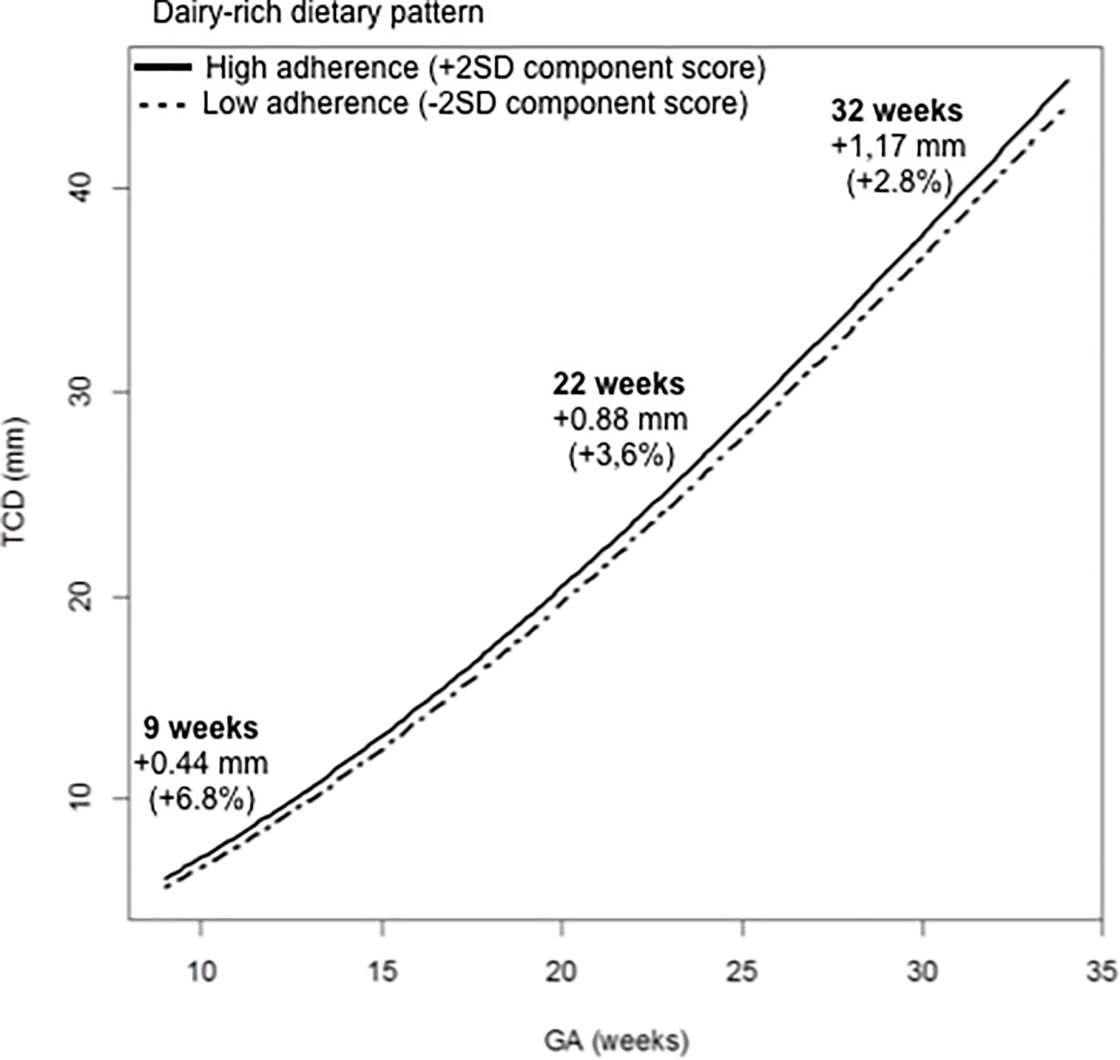
Associations between the adherence to the periconceptional maternal dairy-rich dietary pattern and longitudinal transcerebellar diameter (TCD) measurements after transformation to the original scale (mm) in the study population using model 2. The adherence to the dietary pattern is expressed as -2 standard deviation (SD) (weak) or +2SD (strong) in component score. Full adjustment, including energy intake, conception mode, alcohol, smoke, parity, age, BMI, folic acid supplement/multivitamin use and ethnicity, was performed. GA: gestational age; CI: confidence interval.

The transformation to the original scale showed that strong adherence to this dietary pattern (defined as +2 standard deviation (SD) in component score) increased TCD by 0.44 mm at 9 weeks (+6.8%), 0.52 mm at 11 weeks (+6%), 0.88 mm at 22 weeks (+3.6%), 1.00 mm at 26 weeks (+3.3%), and 1.17 mm at 32 weeks (+2.8%) compared to weak adherence (-2 SD in component score). Fig 2 shows the average regression lines for the dairy-rich dietary pattern in the study population after transformation to the original scale (model 2).

The subgroup analysis on term newborns with normal intrauterine growth trajectory and birth weight (5^th^– 95^th^ percentile, n = 115) did not materially change the associations, showing increased TCD measurements in association with the dairy-rich dietary pattern for both model 1 (β = 0.02 √mm (95% CI: 0.00; 0.03), p<0.05) and 2 (β = 0.02 √mm (95% CI: 0.00; 0.03), p<0.05).

## Discussion

This study shows a positive association between maternal periconceptional adherence to a dairy-rich dietary pattern and prenatal cerebellar growth trajectories assessed by longitudinal TCD measurements from the first trimester onwards. The positive association was confirmed among the subgroup of term pregnancies with normal intrauterine growth trajectory and birth weight. The effect of maternal strong compared to weak adherence to the dairy-rich dietary pattern was higher in the first trimester than later in pregnancy, possibly meaning that additional factors and exposures occurring in the second half of pregnancy could reduce the impact of an early nutritional exposure on cerebellar growth. TCD measurements and trajectories of the study population (Fig 1) were comparable to the reference curves previously provided for normal pregnancies [22]. Finally, the association with higher micronutrient intakes and lower tHcy concentrations supports the healthy nature of the dairy-rich dietary pattern. This is also supported by baseline characteristics of women who highly adhered to the dairy-rich dietary pattern. In fact, despite we did not perform comparisons among groups with high adherence to different dietary patterns due to the overlap resulting from the PCA method itself, women with strong adherence to the dairy-rich dietary patterns (component score>0) were generally highly educated, often multiparous, older and with a low percentage of smoking habit.

The major strength of our study is the longitudinal assessment of human embryonic and fetal cerebellar size with a median of four scans per pregnancy. In this study we collected maternal FFQs at enrolment in early pregnancy, covering maternal dietary habits during the periconceptional period with the first and most important stages of cerebellar development. We used PCA in order to extract dietary patterns from FFQs, avoiding hypothesis-driven definitions of dietary patterns [17]. In this way, this study provides a more complex and comprehensive evaluation of periconceptional maternal nutrition, without focusing on single nutrient or food group intake. Moreover, we checked dietary pattern validity through micronutrient intake and blood biomarkers. To minimize confounding, we adjusted for maternal confounding factors associated with embryonic and cerebellar growth, showing comparable effects in both crude and adjusted models. Finally, the subgroup analysis on term, non-LBW babies allowed to rule out potential cerebellar injuries leading to cerebellar hypoplasia and impaired growth independently on maternal exposures [23]. Inherent to the observational design of the study, also some limitations need to be addressed. 3D US scans were performed to obtain first trimester TCD measurements. We only included ultrasound datasets where the TCD measurements could be optimally performed, resulting in lower success rate than what would be expected in clinical practice, where suboptimal measurements are also accepted. Despite 3D US provides highly accurate biometry and volumetry of embryonic structures, a good quality scan of a lying and still embryo is needed in order to obtain accurate offline TCD measurements. Moreover, the entire embryo was targeted during scanning, without specifically focusing on the cerebellum and possibly resulting in lower success rate of the outcome measurements. It could be discussed that the moderate success rate of first trimester cerebellar measurements may compromise the longitudinal setting of the study [11, 20]. Nevertheless, 64% of patients had two first trimester measurements, 98% had at least two measurements during the whole study period and 93% three or more measurements, leading to a good modeling of cerebellar growth trajectories. As outcome measurement, a single cerebellar diameter was taken into account as a proxy of cerebellar growth and development. Despite additional length and volume measurements could certainly improve the study method and the detection rate of growth abnormalities, we decided to focus on the standardized and most used cerebellar measurement in clinical settings, increasing the generalizability of our results. Furthermore, TCD still represents the most representative brain measurement in case of maternal exposures leading to cerebellar dysfunction, being for instance an essential part of early strategies for *in utero* detection of fetal alcohol spectrum disorders [24]. As dietary surveys could be prone to bias, we compared the mean FIL of the study population with a PAL cutoff for a sedentary lifestyle of 1.35, reducing the likelihood of underreporting. Despite advantages of PCA, the labeling of dietary patterns and the choice of predefined food groups still remain subjective. As we administered FFQs as early as the first trimester, we cannot exclude subsequent variations in maternal dietary habits possibly impacting late cerebellar growth trajectories. Nevertheless, previous studies showed an overall stability of maternal dietary patterns before and during pregnancy, meaning that an early first trimester nutritional assessment may provide a valid representation of maternal diet over pregnancy [25]. Finally, the setting of this cohort study in a tertiary hospital, with high educational level and high rates of chronic comorbidity, pregnancy complications and folic acid supplement use, could reduce the external validity of our findings. This setting also justifies the high percentage of IVF/ICSI pregnancies in the study population and our decision to include them, as well as preterm and LBW babies, as a reliable and representative picture of a tertiary hospital population. Adjustment for conception mode and additional exclusion of preterm/LBW babies were performed to reduce selection bias, but further research is warranted to confirm the associations among low-risk pregnancies. Finally, our study showed that a dairy-rich dietary pattern is significantly associated with cerebellar growth trajectory in a multi-adjusted model including geographical origin. Despite adjustment, further research should evaluate how a dairy-rich, north European typical, dietary pattern impacts on cerebellar development among large non-Caucasian population.

Our findings are in line with previous results showing significant associations between maternal preconceptional initiation of folic acid supplements and increased first trimester cerebellar growth as a function of CRL (β = 0.26 mm (95%CI = 0.02–0.49), p<0.05) [20]. Here we show a first trimester TCD increase by 0.44 mm in case of periconceptional strong adherence to the dairy-rich dietary pattern, with long-term effects of early nutritional exposures occurring also during the second and third trimesters of pregnancy. Recently, cerebellar growth trajectories have been shown to be significantly associated with maternal preconceptional BMI in the same population [26]. Compared to the reference group, overweight women had 4.6%, 3.5%, 1.2%, 1.0% and 0.7% smaller TCD at 9, 11, 22, 26 and 32 weeks respectively. Here we show a double relative difference in case of low *versus* high adherence to a dairy-rich dietary pattern, including adjustment for preconceptional BMI and energy intake. In a prospective study comparing heavy alcohol pregnant consumers to controls, a significant reduction as high as 14% was shown in a single third trimester routine TCD measurement [27]. To our knowledge, no other data are available on the association between prenatal maternal nutrition and human cerebellar growth.

Maternal nutrition has been widely associated with fetal brain development. Animal models of maternal protein and global nutrient restriction showed volume loss, decreased cortical myelination and increased post-mitotic cell death in the cerebellum, with long-term suboptimal performances on behavioral tasks [28, 29]. Besides proteins, maternal micronutrient intake has also been shown to play an important role in fetal brain development, including vitamins B6, B12, E, A, folate and zinc, all significantly associated with the dairy-rich dietary pattern. Possible mechanisms are direct modulation of cell differentiation, neurotrophic factor expression and neurotransmitter synthesis, as well as regulation of fetal immune system and gut microbiota development [30]. Finally, a recent animal model pointed out distinct neonatal cerebellar DNA methylation patterns and gene expression in association with high maternal folic acid supplement use during pregnancy [31]. As the dairy-rich dietary pattern was associated with higher dietary folate intake providing high amounts of substrates and methyl donors, we speculate that the observed associations may be due to epigenetic modifications impacting gene expression and eventually cerebellar growth. Impaired cerebellar methylation patterns and growth have been detected in *post-mortem* human models of autism disorders, however the clinical implications of small increases in prenatal cerebellar growth trajectories need further investigation [32].

In conclusion, we showed that maternal periconceptional adherence to a dairy-rich dietary pattern is associated with slightly increased prenatal cerebellar growth trajectories assessed by longitudinal TCD measurements. We are aware that additional cerebellar measurements and the investigation of neurodevelopmental outcomes in the offspring will contribute to gain new insights into pathophysiological mechanisms of cerebellar development. In early future, intrauterine programming of the cerebellar epigenome through maternal nutrition and exposures might be relevant for the pathophysiology and prevention of several neurologic and psychiatric disorders.

## Supporting information

S1 Table Maternal baseline characteristics according to dietary pattern high adherence. High adherence to the dietary patters was defined as component score>0. Comparison among group was not performed due to the overlapping between groups as a result of PCA, meaning that a single participant can be highly adherent to two or more dietary patterns at the same time. BMI: body mass index, IVF: in vitro fertilization, ICSI: intracytoplasmic sperm injection.
(DOCX)
