# Supplementary material for: Periconceptional maternal dairy-rich dietary pattern is associated with prenatal cerebellar growth
Source: PLoS One. 2018 May 23;13(5):e0197901. doi: 10.1371/journal.pone.0197901 (PMC5965865; doi:10.1371/journal.pone.0197901)
Supplement: S1 Table — High adherence to the dietary patters was defined as component score>0. Comparison among group was not performed due to the overlapping between groups as a result of PCA, meaning that a single participant can be highly adherent to two or more dietary patterns at the same time. BMI: body mass index, IVF: in vitro fertilization, ICSI: intracytoplasmic sperm injection. (DOCX) [file pone.0197901.s001.docx]

**S1 table. Maternal baseline characteristics according to dietary pattern high adherence.**

| **Maternal characteristics** | **High adherence to dietary pattern** | | | |
| --- | --- | --- | --- | --- |
|  | **Mediterranean**  **(n=51)** | **Western**  **(n=55)** | **Egg-rich**  **(n=53)** | **Dairy-rich (n=56)** |
| Age, y median (range) | 32 (26-44) | 31 (22-45) | 33 (22-40) | 33 (23-45) |
| Nulliparous, n(%) | 13 (26.0) | 17 (29.8) | 14 (26.4) | 11 (20.0) |
| Geographical origin  Western, n(%)  Non Western, n(%) | 39 (78.0)  11 (22.0) | 50 (87.7)  7 (12.3) | 45 (84.9)  8 (15.1) | 47 (85.5)  8 (14.5) |
| Educational level  High, n(%)  Intermediate, n(%)  Low, n(%) | 33 (66.0)  13 (26.0)  4 (8.0) | 20 (35.1)  29 (50.9)  8 (14.0) | 29 (54.7)  20 (37.7)  4 (7.5) | 29 (52.7)  21 (38.2)  5 (9.1) |
| BMI, kg/m^2^  median (range) | 22.9  (16.8-39.7) | 23.4  (17.7-39.7) | 24.3  (17.6-34.9) | 22.9  (17.0-39.7) |
| Alcohol use, n(%) | 19 (38.8) | 19 (33.9) | 17 (32.7) | 22 (40.0) |
| Periconception smoking, n(%) | 4 (8.2) | 14 (24.6) | 10 (18.9) | 4 (7.3) |
| Periconception folic acid supplement use, n(%) | 48 (96.0) | 54 (94.7) | 52 (98.1) | 50 (90.9) |
| Mode of conception IVF/ICSI, n (%) | 16 (31.4) | 15 (25.9) | 16 (30.2) | 17 (30.4) |

High adherence to the dietary patters was defined as component score>0. Comparison among group was not performed due to the overlapping between groups as a result of PCA, meaning that a single participant can be highly adherent to two or more dietary patterns at the same time.

BMI: body mass index, IVF: in vitro fertilization, ICSI: intracytoplasmic sperm injection.
